# *Ephedra alata* fruit extracts: phytochemical screening, anti-proliferative activity and inhibition of DPPH, α-amylase, α-glucosidase, and lipase enzymes

**DOI:** 10.1186/s13065-021-00768-9

**Published:** 2021-06-26

**Authors:** Nidal Jaradat, Hanaa Dacca, Mohammed Hawash, Murad N. Abualhasan

**Affiliations:** Department of Pharmacy, Faculty of Medicine and Health Sciences, An-Najah National University, P.O. Box 7, 00970 Nablus, State of Palestine

**Keywords:** Phytochemical, *Ephedra alata*, DPPH, α-Amylase, α-Glucosidase, Lipase, Cytotoxicity

## Abstract

**Background:**

Discovering and screening for potential anti-obesity, anti-diabetic, anticancer, and antioxidant treatments from natural products still in recent times the main goal for many pharmaceutical scientists. The present investigation aimed to evaluate the chemical constituents of *Ephedra alata* fruits various extracts and to assess their antioxidant, antiobesity, antidiabetic, and cytotoxic effects.

**Result:**

In this work, high content of flavonoids and phenols were observed in the methanol fraction of *E. alata* fruits, which reached 98.95 mg of RUE/g and 33.22 mg of GAE/g, respectively. The methanol fraction has significant inhibitory activity against DPPH, α-amylase, α-glucosidase, and lipase with an IC_50_ value of 1.07, 9.43, 46.16, and 66.48 µg/mL. respectively. Also has anticancer activity against HeLa cancer cell line. While the acetone fraction has potent antioxidant activity with IC_50_ 5 µg/mL.

**Conclusion:**

The DPPH and digestive enzymes assays results showed that the methanolic fraction of *E. alata* fruits has potent antioxidant, anti-diabetic, and anti-obesity activities, which can be an excellent candidate for biological and chemical analysis and can be further subjected for isolation of the therapeutically active compounds with anticancer potency.

## Introduction

**Fig. 1:**
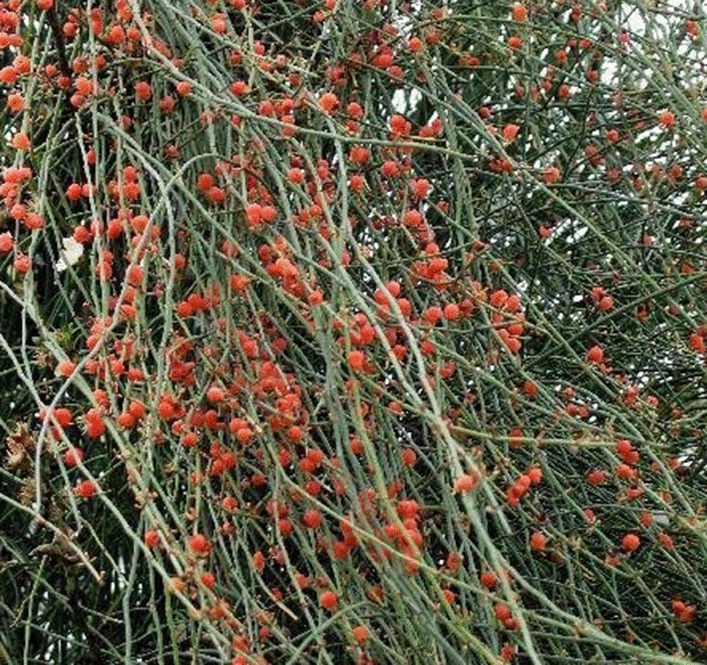
*Ephedra alata* plant red fruits

Most of the population still depends on natural remedies, especially in developing countries. In the past, ancient civilizations depended on local flora and fauna for their survival needs. Despite the fact that some preparations possibly caused harmful effects, or worked by a ceremonial or placebo effect, traditional healing formulations usually had a substantial active pharmacopeia [1]. *Ephedra alata* Decne. (*E. alata*), also known as Alanda in Arabic, is a member of the *Ephedraceae* family. This subspecies is a perennial genus that can exceed more than one meter in height, has a strong pine odor and an astringent taste, belongs to the Gnetales plant, and is the closest living relative of the angiosperm. This ephedra subspecies is native to Iran, Algeria, Egypt, Palestine, Lebanon, Jordan, Iraq, Saudi Arabia, Morocco, Libya, and Tunisia [2, 3]. This plant has a light green, densely branched dioecious, small and perennial stiff shrub, about 50–100 cm tall, the twigs appear leafless and the leaves are reduced to small scales, the cones are sessile shaped and clustered in the axils or at branch tips (Fig. 1) [4]*. Ephedra alata* was globally used in folk medicine, especially its stems, as a decoction of these stems were used as a stimulant, treatment for kidney health problems, bronchial asthma, circular system disorders, and digestive system disturbances, as well as for treatment of cancer. Also, the plant stems are chewed to treat bacterial and fungal infections—especially in oral bacterial and fungal infections [5, 6].

The decongestant effect of *E. alata* stems resulted in its widespread use in medicine for the treatment of cough and sinusitis. In Palestine, *E. alata* has been used in folk medicine to treat the common cold, hay fever, asthma, and in the last few years, it was extensively used for the treatment of cancer [7]. Furthermore, the different extracts of *E. alata* were used as depurative, hypotensive, astringent, and anti-asthmatic natural products. The branches of *E. alata* also seemed to be masticated for cephalalgia, used in miscarriage, and as a bronchodilator, antifungal, and antimicrobial natural agents [8].

The pharmacological effect of the different *Ephedra* species depends on the phytoconstituents of each one. As shown in general studies, the *Ephedra* species were characterized by the alkaloids and phenolic compounds content, such as trans-cinnamic acid, catechin, epicatechin, symplocoside, and flavonol-3-*O*-glycosides, and proanthocyanidins [9].

Oxidation is a process that occurs within the human body that causes damage to cell membranes and other structures, including cellular proteins, lipids, and DNA molecules. When oxygen is rapidly metabolized, it will produce unstable molecules called free radicals that usually steal electrons from other molecules, resulting in DNA damage and other body cells. However, the damage caused by an excessive upload of free radicals over time may become irreversible and lead to certain dangerous diseases, including heart disease, liver disease, and some types of cancers, especially oral, stomach, esophageal, and bowel cancer [10].

According to data collected from the World Health Organization (WHO) global survey on traditional, complementary/alternative, and herbal medicines, the market for these kinds of medicines is steadily growing worldwide. In actuality, the utilization of phytopharmaceuticals and nutraceutical products͑ is continuously expanding. Nowadays, many people have been using these natural formulations in the treatment or prevention of various diseases and health disorders in different national healthcare centers [11, 12].

The worldwide incidence of diabetes is increasing every year. Based on information from the International Diabetes Federation the estimated number of people with diabetes reached 30 million͑ in 1985, 150 million in 2000, and 246 million in 2007͑. They also expect that the number of diabetic patients may hit 380 million by 2025 [13]. This health problem prevails worldwide with its occurrence rising at an alarming rate all over the world. Different complications encompass all the vital organs of the human body as a consequence of the metabolic derangement in diabetes [14, 15].

Cancer is a condition that refers to different types of diseases that are typically characterized by rapid and abnormal growth of human cells beyond the usual boundaries—proliferation cannot be controlled and cells acquire anti-apoptosis features giving them the ability to penetrate and destroy the normal body tissue. Also, cancer can spread and affect any organ or part of the human body [16]. The development process for cancer drugs has relied on natural products where more than 70% of the available anti-cancer drugs are from natural resources, and plants are most often used. However, more than 3000 plants in the world have been reported and known to have anti-cancer properties [17].

The objective of the present study is to investigate the chemical composition of *E. alata* various extract and to correlate the chemical profile with the antioxidant activity, pancreatic lipase inhibitory, carbohydrate-hydrolyzing enzymes, and anticancer properties.

## Materials and methods

### Chemicals and reagents

The following materials were purchased from Frutarom (Israel), Sigma-Aldrich (Germany, USA, and Denmark), Riedel–de Haen (Germany), Alfa-Aesar (England) and Loba/Chemie, Merck, SDFCL (India): Ferric chloride, sulfuric acid (H_2_SO_4_), methanol, iodine, Benedict’s solution, hexane, acetone, chloroform, Molisch’s reagent, Trolox ((s)-(-)-6 hydroxy-2,5,7,8-tetramethychroman-2-carboxylic acid, Acarbose, α-glucosidase (Baker’s Yeast alpha-glucosidase), α-amylase, 3,5-dinitrosalicylic acid (DNSA), potassium phosphate, Gallic acid, sodium carbonate, sodium hydroxide (NaOH), hydrochloric acid (HCl), Ninhydrin reagent, magnesium ribbon, Folin-Ciocalteu’s reagent, L-glutamine solution, MTS reagent, phosphate-buffered saline, a Pen-Strep solution composed of penicillin and streptomycin, 2,2-Diphenyl-1-picrylhydrazyl (DPPH), porcine pancreatic amylase enzyme solution, and starch.

### Samples and extraction procedures

The *E. alata* fruits were collected from the Jenin area of Palestinian sites in July of 2018. Identifications were performed by the pharmacognosist Dr. Nidal Jaradat, at the Pharmacognosy Laboratory at An-Najah National University-Nablus Palestine (voucher series: Pharm-PCT-904). The fruits were washed and cleaned two times with distilled water and dried in the oven at 40 ºC. Finally, the fruits were grounded into powder by the mechanical grinder and kept in a well-closed container with a suitable label for further use.

The extraction process adopted in this research was based on the fractional extraction procedure, which conducted by adding different solvents sequentially based on their polarity, starting with the most nonpolar solvent, hexane, followed by acetone (polar aprotic organic solvent), then methanol-a highly polar, low molecular weight alcohol-and finishing the extraction steps with distilled water (polar inorganic solvent). For the preparation of each extract fraction, 25 g of the dried, grounded fruits were first soaked in 0.5 L of hexane for 72 h (three days) in a shaker device, with continuous shaking at approximately 100 rounds/minute at room temperature. Then, the hexane was replaced with 0.5 L of acetone at the same conditions mentioned above, followed by methanol and finally distilled water. Each organic fraction was filtered using suction filtration and concentrated under a vacuum on a rotary evaporator, while the aqueous fraction was collected as a powder using a freeze dryer. All crude fractions were kept at 4 °C for further use [18].

The yield of each extract fraction was calculated using the following formula:

$$ \% {\text{ Yield }} = {\text{ }}\left( {{\text{Weight of}}\,E.{\text{ }}alatafruits{\text{ extract}}/{\text{weight of dry fruits}}} \right){\text{ }} \times {\text{ }}100\% $$

### Determination of total phenol content (TPC)

The content of total phenolic compounds of *E. alata* fruits for four different extracts was assessed using the Folin-Ciocalteu’s reagent (FCR). FCR, or Folin's phenol reagent, is a mixture of phosphomolybdate and phosphotungstate used for the colorimetric assay of phenolic and polyphenolic antioxidants.

Using a 100 mL volumetric flask, a 7.5% sodium carbonate (Na_2_CO_3_) solution was prepared by dissolving 7.5 g of Na_2_CO_3_ in less than 100 mL of distilled water, then, distilled water was used to bring the volume up to 100 mL. Similarly, to sodium carbonate solution, a stock solution of the standard solution (Gallic acid solution) was prepared by dissolving 100 mg of gallic acid and dissolved up to 100 mL of distilled water. The reaction mixture was prepared by mixing 0.5 mL of each extract solution, 2.5 mL of 10% Folin-Ciocalteu’s reagent dissolved in water, and 2.5 mL of 7.5% sodium carbonate (Na_2_CO_3_) in a test tube for each sample. The sample tubes were incubated for 45 min at 45 ºC. The absorbance was determined using a spectrophotometer at wavelength 765 nm. The samples were prepared in triplicate for each analytic trial to obtain the mean and standard deviation values [19].

### Determination of total flavonoid content (TFC)

Total flavonoid content (TFC) was determined according to the procedure adopted by Chang et al. The TFC was calculated from the calibration curve of Rutin (the used standard) and expressed as the milligram of Rutin Equivalent per gram of extract (mg RUE/g extract).

The calibration curve for Rutin was established using serial dilutions, starting with the preparation of a stock solution of 100 µg/mL-10 mg of Rutin was dissolved in 10 mL of distilled water and diluted to 100 mL. Subsequently, the stock solution was diluted to provide a series of concentrations of 10, 30, 40, 50, 70, and 100 µg/mL.

For the preparation of the working solutions, 0.5 mL of each solution was mixed with 3 mL methanol, 0.2 mL of 10% AlCl_3_, 0.2 mL of 1 M potassium acetate, and 5 mL of distilled water, and then, incubated at room temperature for 30 min. The previous steps were performed for each fruit extract fraction, and finally, the absorbance was recorded at a 415 nm wavelength, and distilled water with methanol, 10% AlCl_3,_ and potassium acetate was used as a blank solution. The samples were prepared in triplicate for each analytic trial to obtain the mean and standard deviation values [20].

### Determination of total tannin content (TTC)

For total tannin content assessment, according to the method of Sun et al., the four *E. alata* fruit extract fractions. Catechin was used as a reference compound to construct the calibration curve for the needed calculations. A 100 µg/mL stock methanolic solution was prepared, and then, serial dilutions were obtained (10, 30, 50, 70, and 100 µg/mL).

A 4% methanolic vanillin solution was prepared freshly. 100 µg/mL stock solution from each fruit extract fraction was prepared using methanol as a solvent. For the working solution, each test tube contained 0.5 mL from each extract mixed with 3 mL of vanillin solution and 1.5 mL of concentrated HCl. The mixture was allowed to stand for 15 min, and then the absorption was measured at 500 nm against methanolic vanillin as a blank. All working samples were analyzed in triplicate. The total tannin in each fraction was expressed as catechin equivalents (mg of CAE/g of plant fraction) [21].

### DPPH radical scavenging activity assay

The *E. alata* fruit extract stock solutions were serially diluted to achieve concentrations of 100, 50 20, 10, 5, and 2 µg/mL using methanol as solvent. Each test tube contained 1 mL of each concentration and was marked properly. One mL of 0.002% methanolic DPPH solution was added to each test tube, and 1 mL of methanol was added to each test tube to bring the final volume up to 3 mL (caution: DPPH is light sensitive, so preparation of working test tubes should be performed with minimum light exposure).

The samples were incubated for 30 min in a dark place, then, their optical densities were determined using the spectrophotometric measurement at a wavelength of 517 nm. The equation used in this analytical study to calculate the inhibition percentage is shown below:

$$ \% {\text{ DPPH inhibition }} = {\text{ }}\left( {{\text{A}}_{{\text{B}}}  - {\text{A}}_{{\text{E}}} } \right)/{\text{A}}_{{\text{B}}}  \times {\text{1}}00\% $$

A_B_ is the recorded absorbance of the blank solution; A_E_ is the recorded absorbance of the *E. alata* sample solution [22].

### Porcine pancreatic lipase inhibition assay

The porcine pancreatic lipase inhibitory method was followed in this study according to protocols from Zheng et al. and Bustanji et al., with some modifications [23]. A stock solution of 500 µg/mL from each plant fraction, in 10% DMSO, was used to prepare five different solutions with the following concentrations: 50, 100, 200, 300, and 400 μg/mL. A 1 mg/mL stock solution of porcine pancreatic lipase enzyme was freshly prepared in Tris–HCl buffer before use. The substrate used for this study, *p*-nitrophenyl butyrate (PNPB), was prepared by dissolving 20.9 mg in 2 mL of acetonitrile. For each working test tube, 0.1 mL of porcine pancreatic lipase (1 mg/mL) was mixed with 0.2 mL of each diluted solution series for each plant fraction. The resulting mixture was then brought to a total volume of 1 mL, by adding Tri-HCl solution and incubated at 37 °C for 15 min. Following the incubation period, 0.1 mL of PNPB solution was added to each test tube. The mixture was incubated for 30 min at 37 °C. Pancreatic lipase activity was determined by measuring the hydrolysis of the PNPB compound into *p*-nitrophenolate ions at 410 nm using a UV spectrophotometer. The same procedure was repeated for Orlistat, which was used as a standard reference compound. The equation used in this analytical study is shown below:

$$ \% {\text{ Lipase inhibition }} = {\text{ }}\left( {{\text{A}}_{{\text{B}}}  - {\text{A}}_{{\text{E}}} } \right)/{\text{A}}_{{\text{B}}}  \times {\text{1}}00\% $$

A_B_ is the recorded absorbance of the blank solution; A_E_ is the recorded absorbance of the *E. alata* sample solution.

### α-Amylase inhibition assay

Each extract fraction was dissolved in a few milliliters of 10% DMSO and then further dissolved in buffer (0.02 M of Na_2_HPO_4_/NaH_2_PO_4_, 0.006 M NaCl, at pH 6.9) to give concentrations of 1000 μg/mL, from which the following dilutions were prepared: 10, 50, 70, 100, 500 μg/mL. The porcine pancreatic α-amylase enzyme solution was freshly prepared at a concentration of 2 units/mL in 10% DMSO.

For working solutions, a volume of 0.2 mL of enzyme solution was mixed with 0.2 mL of each *E. alata* fruit extract fraction and was incubated for 10 min at 30 °C. After the incubation period, 0.2 mL of a freshly prepared 1% starch aqueous solution was added to each working solution, followed by an incubation period of at least 3 min. The reaction was stopped by the addition of 0.2 mL dinitrosalicylic acid (DNSA) yellow color reagent. Each working solution was then diluted with 5 mL of distilled water and then boiled for 10 min in a water bath at 90 °C. The mixture was cooled to room temperature, and the absorbance was taken at 540 nm. The blank was prepared following the same steps above, but the plant fraction was replaced with 0.2 mL of the previously described buffer. Acarbose was used as the standard reference following the same steps used for plant extract fractions [24].

The α-amylase inhibitory activity was calculated using the following equation:

$$ \% {\text{ of }}\alpha  - {\text{amylase inhibition }} = {\text{ }}\left( {{\text{A}}_{{\text{B}}} {-}{\text{A}}_{{\text{E}}} } \right)/{\text{ A}}_{{\text{B}}}  \times {\text{1}}00\% $$

A_B_: is the absorbance of blank; A_E_: is the absorbance of *E. alata* sample.

### α-Glucosidase inhibition assay

The enzyme, alpha-glucosidase (1 U/mL), and 20 μL of different concentrations of each extract fraction (100, 200, 300, 400, and 500 mg/mL) were added to a test tube. In each working test tube, a reaction mixture contained 0.1 mL of alpha-glucosidase solution was mixed with 0.2 mL from each extract dilution and 0.5 mL of phosphate buffer (100 mM, pH = 6. 8). The samples were incubated at nearly 37 °C for 15 min. After this incubation period, 0.2 mL of 5 mM PNPG (the substrate used for this experiment) was added to the reaction mixture, and the samples were again incubated at 37 °C for 20 min. The reaction was terminated by adding 0.1 M sodium carbonate (Na_2_CO_3_). The absorbance at the 405 nm wavelength was recorded for all samples. Acarbose was used as a positive control at the same concentrations as the plant extracts [25]. The results were expressed as percentage inhibition according to the following equation:

$$ \alpha {-}{\text{Glucosidase inhibition }}\left( \%  \right){\text{ }} = {\text{ }}\left( {{\text{A}}_{{\text{B}}}  - {\text{ A}}_{{\text{E}}} /{\text{A}}_{{\text{B}}} } \right){\text{ }} \times {\text{1}}00\% $$

A_B_ is the absorbance without enzyme inhibitor; A_E_ is the absorbance in the presence of *E. alata* sample.

### MTS assay

HeLa cervical adenocarcinoma cells were cultured in RPMI-1640 media supplemented with 10% fetal bovine serum, 1% Penicillin/Streptomycin antibiotics, and 1% L-glutamine. The cells were grown in a humidified atmosphere with 5% CO_2_ at 37 °C. The cells were seeded at 2.6 × 10^4^ cells/well in a 96-well plate. After 48 h, the cells were incubated with various concentrations of the tested compounds for 24 h. Cell viability was assessed by Cell Tilter 96® Aqueous One Solution Cell Proliferation (MTS) Assay according to the manufacturer’s instructions (Promega Corporation, Madison, WI, USA). Briefly, at the end of the treatment, 20 μL of MTS solution per 100 μL of media was added to each well and incubated at 37 °C for 2 h. The absorbance was measured at 490 nm [26].

### Statistical analysis

All of the obtained results of the four studied *E. alata* fruit fractions were expressed as mean ± SD standard deviation and the results were considered significant when the *p-*value was < 0.05. The percentage contents of *E. alata* plant and the bioactivity of its extracted samples were subjected to principal component analysis (PCA) and partial least-squares discriminant analysis (PLS-DA) with the aid of SIMCA software 14.1. Score scatter plot and Loading scatter plot were generated. The X-axis and Y-axis represent score vectors summarizing all the variables entering the analysis.

## Results and discussion

### Phytochemical screening

After performing the previously mentioned phytochemical laboratory tests, it was observed that the different *E. alata* fruit extract fractions contained a variety of active phytochemical ingredients, which were summarized in Table 1. It was observed that the alternative fractionation process, adding different solvents in this extraction method, was effective in separating the active phytoconstituents from each other. Proteins, saponin, reducing sugars, and starches were collected intensively in the aqueous fraction, while alkaloids appeared in all extract fractions. Flavonoids, which are always resembled anti-oxidant activity, appeared in all extract fractions, and they were found in high quantity in the methanol layer. Phenols and tannins were observed in all fractions but seemed to be concentrated in the methanol layer. Positive results for volatile oil were found in the methanol fraction, confirming that the presence of one of the alcohol-type volatile oils.

**Table 1:** Phytochemical screening tests for different extract fractions of *E. alata* fruits

Phytochemical active constituent	Hexane extract	Acetone extract	Methanol extract	Aqueous extract
Protein & amino acids Biuret test	−	−	+	+
Reducing sugars Fehling’s test	−	−	−	+
Complex polysaccharides Molisch’s test	+	+	–	−
Starch Iodine test	−	−	−	+
Phenols Ferric chloride test	+	+	+	+
Tannins Gelatin test	+	+	+	+
Flavonoids Shinoda reagent	−	+	+	+
Saponin Foam test	−	−	−	+
Glycosides Keller-Killani Test	−	−	−	−
Steroids	+	−	−	−
Terpenoids Salkowski’s test	+	−	−	−
Alkaloids Wagner’s test	+	+	+	+
Volatile oil KOH test	−	−	+	−

Where ( +) means the presence of phytochemicals and (−) absence of phytochemicals

As shown in Table 2, the percent yield results recorded after calculations of each extract fraction revealed that the highest yield was observed in the methanolic fraction, which was 29%, followed by the acetone and aqueous fractions, which were 15.6% and 15.08%, respectively.

**Table 2:** The yield percentage for four extract fractions of *E. alata* fruits

Extract Fractions	Extract (g)	Yields (%)
Hexane	2.15 g	8.6%
Acetone	3.9 g	15.6%
Methanol	7.25 g	29%
Aqueous	3.77 g	15.08%

### Quantitative analysis of TPC, TFC, and TTC

For the evaluation of TPC, TFC, and TTC, the absorption (Abs) values of several concentrations of the Gallic acid, Rutin, and Catechin standards (STDs) are shown in Table 3, and regarding these points, three equations were obtained for each STD versus its concentrations to calculate the total phenol, flavonoid, and tannin contents of the hexane, acetone, methanol, and aqueous *E. alata* fruits fractions are presented in Table 4.

**Table 3:** Absorbance values of the STDs at different concentrations

Conc. of STDs and λmax					
Conc. of Gallic acid (μg/mL)	0	10	40	50	70
Abs. at λmax = 765 nm	0	0.142	0.496	0.557	0.798
Conc. of Rutin (μg/mL)	0	10	30	50	60
Abs. at λmax = 415 nm	0	0.049	0.11	0.17	0.2
Conc. of Catechin (μg/mL)	0	10	30	50	70
Abs. at λ_max_ = 500 nm	0	0.028	0.041	0.056	0.077

**Table 4:** Quantitation of phenols, tannins, and flavonoids in hexane, acetone, methanol, and aqueous fractions of *E. alata* fruits

*E. alata* fruit extract fractions	Total flavonoids contents, mg of RUE/g of dry extract ± SD	Total phenol contents, mg of GAE/g of dry extract ± SD	Total tannin contents, mg of CAE/g of dry extract ± SD
Hexane	–	5.72 ± 0.39	2.5 ± 0.70
Acetone	58.95 ± 2.33	19.85 ± 1.62	10.5 ± 0.70
Methanol	98.95 ± 2.3	33.22 ± 1.56	17.5 ± 0.70
Aqueous	32.3 ± 2.4	25.9 ± 0.78	1.5 ± 0.70

### Antioxidant activity

**Fig. 2:**
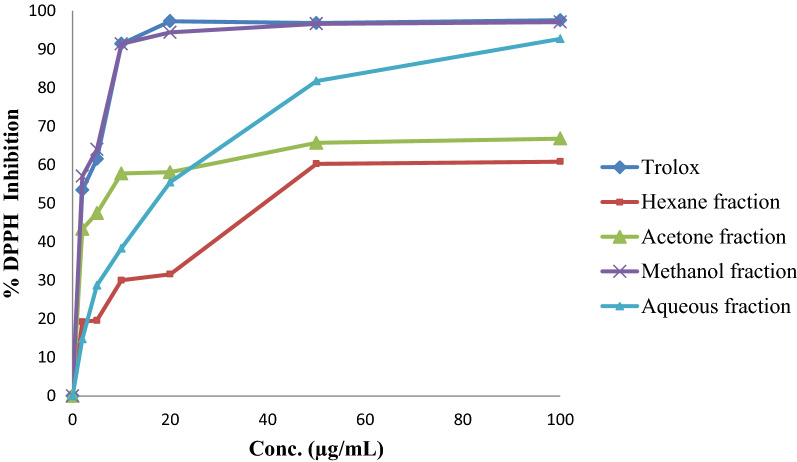
The DPPH inhibition percentage of the different *E. alata* fruit extract fractions compared to Trolox (positive control)

The results of assessing the free radical scavenging activity of four fractions from *E. alata fruits*, using Trolox as a reference antioxidant agent, were expressed as percentage DPPH inhibition (Fig. 2 and Table 5). Therefore, *E. alata fruits* could be considered an herbal source for antioxidants, especially for the methanol fraction, which had an IC_50_ value of 1.70 ± 0.25 µg/mL. Similar results were also obtained for the acetone fraction, which showed an IC_50_ value of 5.00 ± 0.51 µg/mL. The results were compared to Trolox, a potent antioxidant compound, with an IC_50_ equal to 2.04 ± 0.74 µg/mL. By contrast, the aqueous fraction only showed moderate antioxidant activity, with a higher IC_50_ value of 15.25 ± 0.30 µg/mL.

**Table 5:** The IC_50_ for different extracts fractions against DPPH, Lipase, α‑Amylase, and α-glucosidase in comparison of IC_50_ of positive controls

	Target enzymes	Reference	Hexane fraction	Acetone fraction	Methanol fraction	Aqueous fraction
IC_50_ (µg/mL)	DPPH	2.04 ± 0.74^a^	44.05 ± 0.39	5.00 ± 0.51	1.70 ± 0.25	15.25 ± 0.30
	Lipase	12.3 ± 0.33^b^	277.25 ± 0.79	77.56 ± 0.34	66.48 ± 0.50	274.407 ± 0.49
	α‑Amylase	28.84 ± 1.22^c^	55.01 ± 1.23	189.94 ± 2.32	9.43 ± 0.6	16.37 ± 0.58
	α-Glucosidase	37.15 ± 0.33^C^	167.68 ± 0.38	NI	46.16 ± 0.63	201.77 ± 0.48

^a^ Trolox, ^b^ Orlistat, ^c^ Acarbose, NI: no inhibition (inhibition at concentration higher than 400 µg/mL)

These observations confirmed the results of the quantitative analyses of phenols, flavonoids, and tannins, which showed a high flavonoid and phenol content in the methanol extract fraction, which was equal to 98.95 ± 2.3 mg of RUE/g of dry extract and 33.22 ± 1.56 mg of GAE/g of dry extract, respectively. Tannins also showed the highest content in the methanol fraction with a value equal to 17.5 ± 0.70 mg of CAE/g of dry extract. In general, the phenolic content of all the extracts was considerably high, which could be a major contributor to the strong antioxidant effect of *E. alata* fruit extracts. Therefore, the high phenolic content in fruit extracts will explain these results of high antioxidant activity [27].

### Lipase inhibition activity

**Fig. 3:**
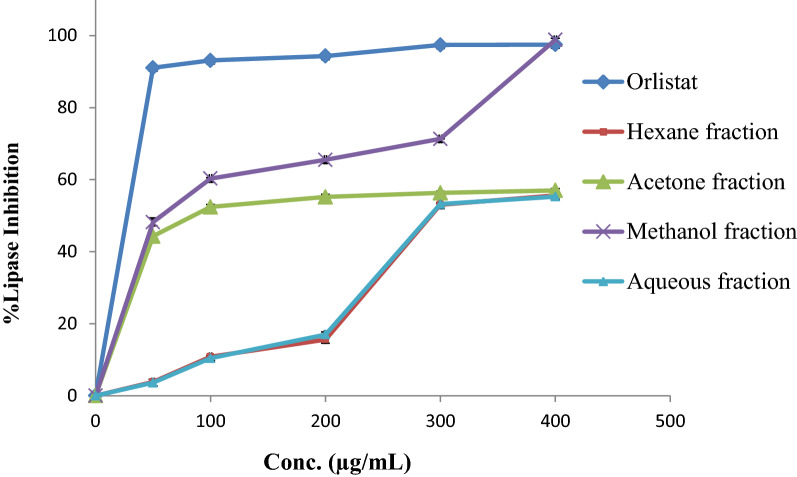
The lipase inhibition percentage of the different *E. alata* fruit extract fractions compared to Orlistat (positive control)

In this assay, the anti-obesity activity of fractions from *E. alata fruits* extracts was compared to that of orlistat, a positive control (Fig. 3 and Table 5). *E. alata fruits* were an excellent alternative natural source of lipase inhibitory agents. The methanol fraction showed an IC_50_ value of 66.48 ± 0.50 µg/mL, which was the most potent fraction in comparison with the reference compound orlistat (12.3 ± 0.33 µg/mL). However, acetone fraction showed a similar inhibition profile as methanol, while hexane and aqueous fractions were showed weak inhibitory activity with IC_50_ values 77.56, 277.25, and 274.407 µg/mL respectively. These observations confirmed the results of the quantitative analyses of phenols, flavonoids, and tannins that showed a high content of flavonoids and phenols in the methanol extract fraction mentioned before.

### α‑Amylase inhibition activity

**Fig. 4:**
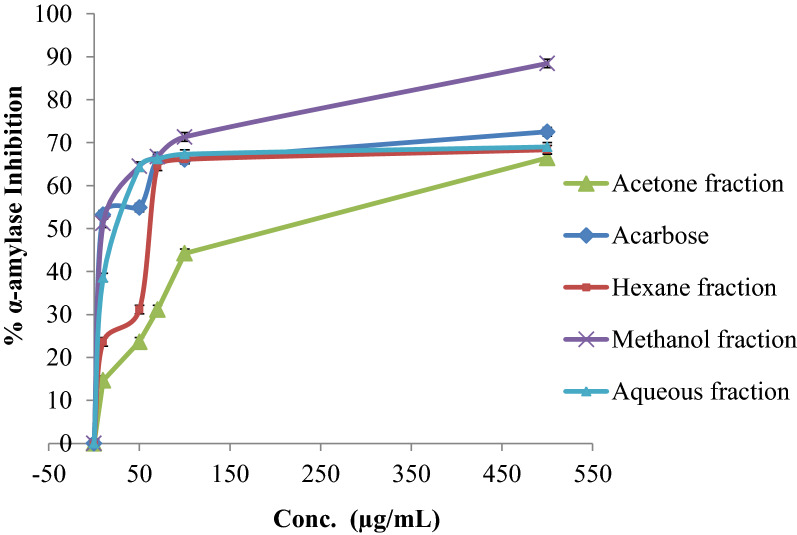
α-Amylase inhibition percentage of the different *E. alata* fruit extract fractions compared to Acarbose (positive control)

In this assay, the α-amylase inhibitory activity of fractions from *E. alata* fruits extract was compared to the positive control Acarbose (Fig. 4). The methanol fraction was the most potent inhibitor of α-amylase, with an IC_50_ of 9.43 ± 0.6 µg/mL, compared to 28.84 ± 1.22 µg/mL for acarbose, the reference compound, and the aqueous fraction has similar activity with IC_50_ 16.37 ± 0.58 µg/mL. in contrast, the other fractions have weaker activities against this enzyme (Table 5). The results obtained in this research, especially for the methanol fraction which possessed the highest phenolic content, provide additional evidence that is in line with previous studies that natural polyphenols can inhibit the activity of carbohydrate hydrolyzing enzymes like α-amylase and α-glucosidase [28].

### α-Glucosidase inhibition activity

**Fig. 5:**
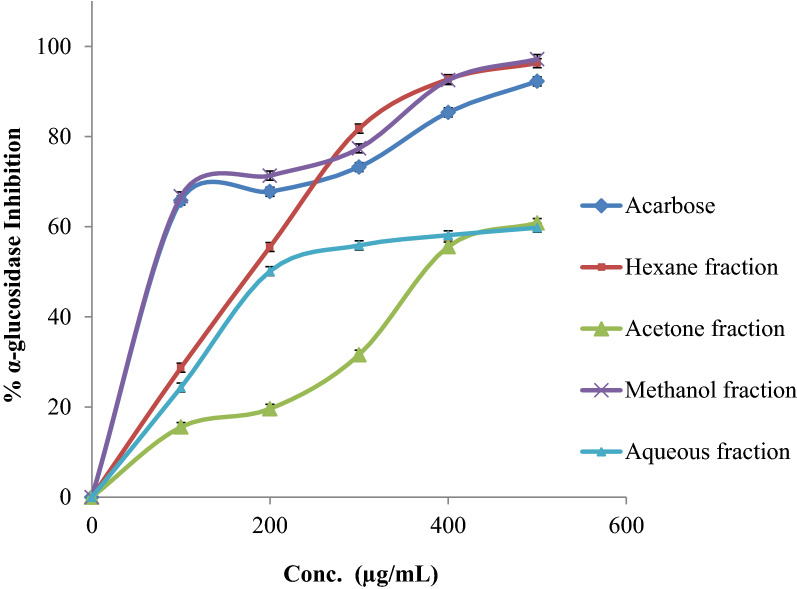
α-Glucosidase inhibition percentage of the different *E. alata* fruit extract fractions compared to Acarbose (positive control)

Results of α-glucosidase were compared with those of acarbose, positive control, and the IC_50_ values were calculated for the four fractions (Table 5 and Fig. 5). The methanol fraction exerted the greatest inhibitory action on α-glucosidase with an IC_50_ of 46.16 ± 0.63 µg/mL, compared with that of acarbose, the reference control, at 37.15 ± 0.33 µg/mL. In contrast, the other fractions showed moderate to negligible inhibition of α-glucosidase. The results obtained in this study confirmed previously observed results that revealed the activity of phenolic compounds like *p*-hydroxybenzoic acid, as well as *trans-p*-coumaric acid and epicatechin gallate, and flavonoids, like quercetin that are present in lentil extracts, to be effective inhibitors of some digestive enzymes lipase and α-glycosidase contributing to controlling glucose levels in the blood, as well as the management of obesity [29].

### Anti-proliferative activity

**Fig. 6:**
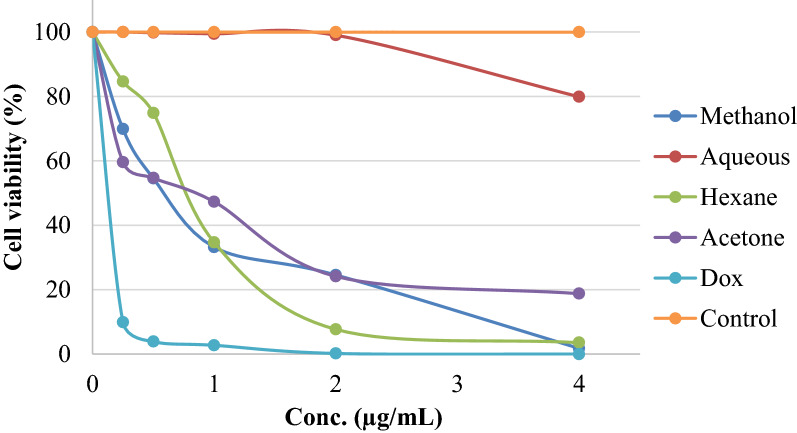
Effects of the fractions in comparison with negative control (untreated) and positive control (Dox) on HeLa Cell Viability

The results of treatment of HeLa cancer cells with five different concentrations in mg/mL for different fractions showed that methanol, acetone, and hexane fractions have cytotoxic activities with IC_50_ range 604–764 µg/mL while the aqueous fraction has no activity. The cell viability graph (Fig. 6) showed the activity of these fractions in comparison with negative control and positive control (Doxorubicin). In previous studies on cytotoxicity of the hydroalcoholic extract of the aerial part of *E. alata*, the results showed that the extract contained polyphenolic phyto-compounds and had anti-proliferative, pro-apoptotic, and cytotoxic potential against the MCF-7 human breast cancer cell line [30]. But in our study, the anticancer activity can be considered as very weak in comparison with anticancer drugs such as the used positive control Doxorubicin.

### Principal component analysis (PCA)

**Fig. 7:**
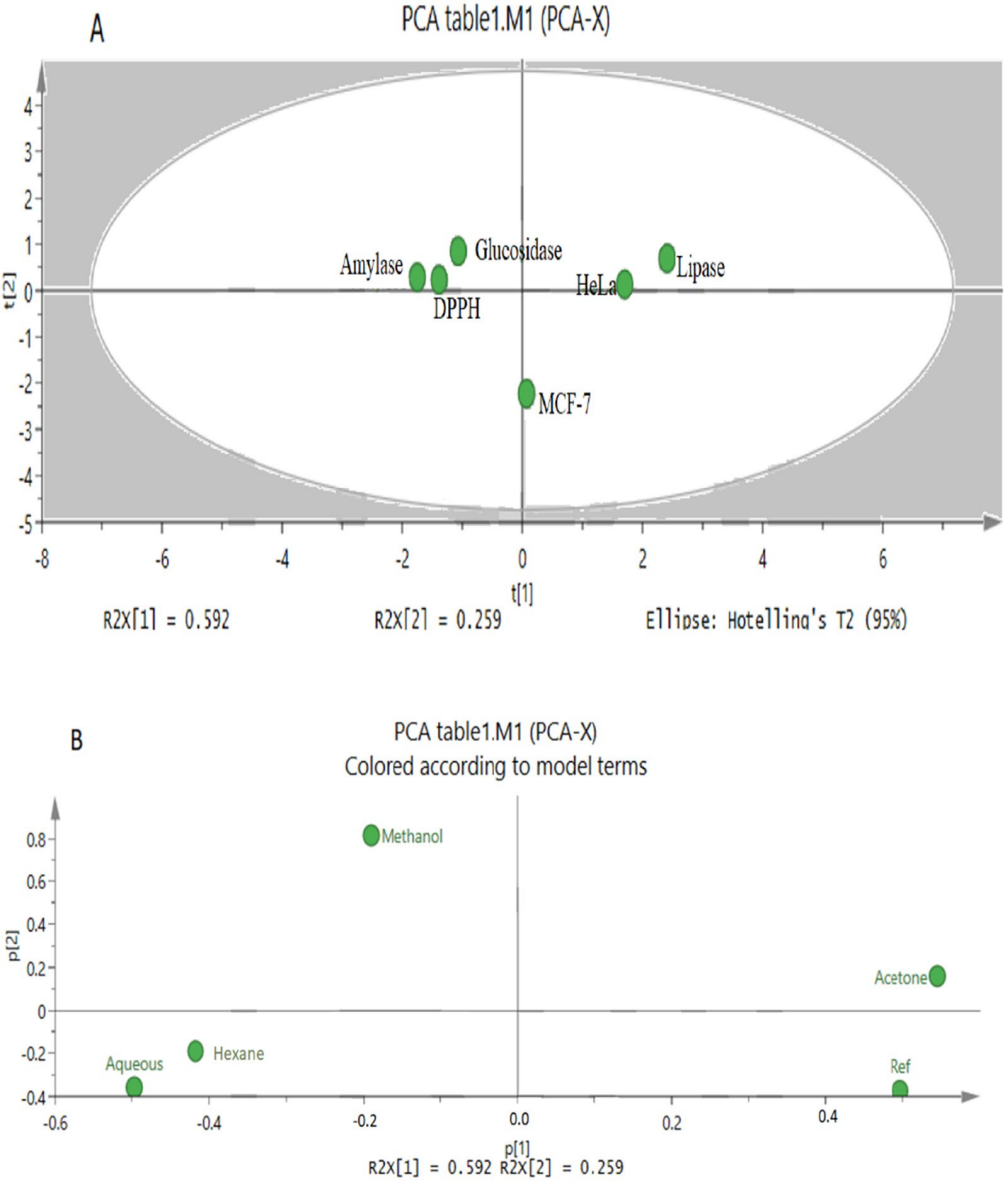
PCA results of *E. alata* fruit bioactivity inhibition results using different solvents **A** Score scatter plot **B** Loading scatter plot

**Fig. 8:**
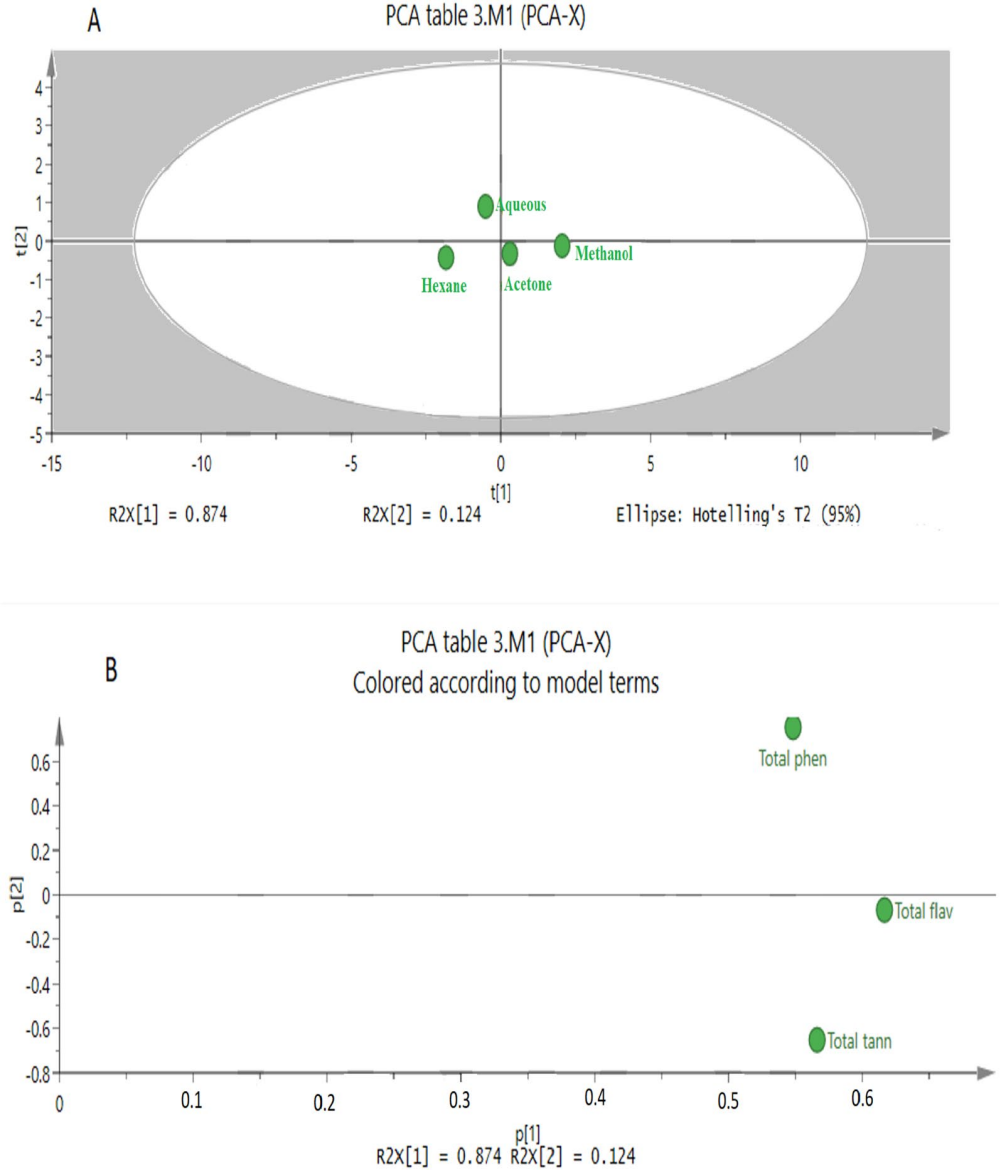
PCA results of *E. alata* fruit chemical constituents using different solvents **A** Score scatter plot **B** Loading scatter plot

Principal component analysis (PCA) is the statistical tool which was used to discuss the variations between different samples and to find more data on the variables which influence the sample similarities and differences [31]. The score plot (Fig. 7A) shows that the product has similarity of inhibition activities for amylase, DPPH and glucosidase. Theses bioactivities were opposite in terms of potency for lipase and cytotoxicity of HeLa cells. However, the cytotoxicity against MCF-7 is similar regardless of the extracting solvent. Loading scatter plot (Fig. 7B) shows that solvent methanol has the largest absolute loading values. The chemical constituents of the plant were also analyzed using the SIMCA program. The generated Score scatter plot Fig. 8A demonstrate that Hexane solvent has an extraction capability opposite to that of methanol while the aqueous and acetone solvent were we not distinguishable. The loading scatter plot Fig. 8B shows that total phenol has the largest absolute loading values. Regarding the data obtained from PCA, there is a clear relationship between the activities of methanol fraction as the most active fraction towered various biological targets and the highest percentage of TFC, TPC, TTC in this fraction.

## Conclusion

The *E. alata* fruit extracts analysis showed that it contains a mixture of different phytochemicals such as protein, amino acid, reducing sugars, and saponin, in addition to phenols, tannins, and flavonoids which seems to be in high quantity in particular in the methanol extract. The methanol extract has potent antioxidant, α-amylase, and α-glucosidase inhibition, and moderate lipase inhibitory activity. Therefore, the methanol extract fraction of *E. alata* provides a scientific rationale for the use in the pharmaceutical industry as a low-cost nutrient useful in reducing chronic pathologies such as diabetes mellitus, obesity, and oxidative stress. The methanol extract fraction was shown to be a potent inhibitor of starch digestive enzymes due to the high content of phenolic compounds and flavonoids, and it was found to be effective in free radical scavenging and lipase enzyme inhibition. In addition, the methanol extract derived from *E. alata* fruits induced cytotoxicity at a higher concentration by approximately 94%, and therefore, it may represent a good choice for some of the health beneficial herbal supplements and natural medications used in cancer management and can be further subjected for the isolation of the therapeutically active compounds.

## Data Availability

The datasets used and/or analyzed during the current study available from the corresponding author on reasonable request.

## Abbreviations

DPPH: 2, 2-Diphenyl-1-picrylhydrazyl
IC_50_: Half maximal inhibitory concentration
*E. alata*: *Ephedra alata*
PNPG: *p*-Nitrophenyl glucopyranoside
PNPG: *p*-Nitrophenyl glucopyranoside
Trolox: ((S)-(-)-6 hydroxy-2,5,7,8-tetramethychroman-2-carboxylic acid)
A_B_: Absorbance of the blank solution
A_E_: Absorbance of the *E. alata* sample solution
PNPB: *p*-Nitrophenyl butyrate
CAE: Catechin equivalent
GAE: Gallic acid equivalent
RUE: Rutin equivalent
WHO: World Health Organization
DM: Diabetic mellitus
DNSA: 3,5-Dinitrosalicylic acid
TPC: Total phenols content
TFC: Total flavonoid content
TTC: Total tannin content
CA: Principal component analysis

## References

[1] Bensky D, Clavey S, Stõger E (2004). Materia medica.

[2] Alqarawi A, Hashem A, Abd-Allah E, Alshahrani T, Huqail A (2014). Effect of salinity on moisture content, pigment system, and lipid composition in *Ephedra alata* Decne. Acta Biol Hung.

[3] Caveney S, Charlet DA, Freitag H, Maier-Stolte M, Starratt AN (2001). New observations on the secondary chemistry of world *Ephedra* (Ephedraceae). Am J Bot.

[4] Nawwar M, Barakat H, Buddrust J, Linscheidt M (1985). Alkaloidal, lignan and phenolic constituents of Ephedra alata. Phytochemistry.

[5] Al-Qarawi A, Abd Allah E, Hashem A (2012). Effect of Ephedra alata on nucleic acids and nitrogen metabolism of seedborne Aspergillus flavus. Pak J Bot.

[6] Freitag H, Maier-Stolte M (2003). The genus Ephedra in NE tropical Africa. Kew Bull.

[7] Abourashed EA, El-Alfy AT, Khan IA, Walker L (2003). Ephedra in perspective–a current review. Phytother Res.

[8] Ghanem S, El-Magly UI (2008). Antimicrobial activity and tentative identification of active compounds from the medicinal Ephedra alata male plant. J Taibah Univ Med Sci.

[9] Al-Rimawi F, Abu-Lafi S, Abbadi J, Alamarneh AA, Sawahreh RA, Odeh I (2017). Analysis of phenolic and flavonoids of wild Ephedra alata plant extracts by LC/PDA and LC/MS and their antioxidant activity. Afr J Tradit Complement Altern Med.

[10] Jadhav S, Nimbalkar S, Kulkarni A, Madhavi D (1995). Lipid oxidation in biological and food systems. Food antioxidants.

[11] Bustanji Y, Issa A, Mohammad M, Hudaib M, Tawah K, Alkhatib H (2010). Inhibition of hormone sensitive lipase and pancreatic lipase by Rosmarinus officinalis extract and selected phenolic constituents. J Med Plant Res.

[12] Zheng C-D, Duan Y-Q, Gao J-M, Ruan Z-G (2010). Screening for anti-lipase properties of 37 traditional Chinese medicinal herbs. Chin Med J.

[13] Mosleh R, Hawash M, Jarrar Y (2020). The relationships among the organizational factors of a tertiary healthcare center for type 2 diabetic patients in palestine. Endocr Metab Immune Disord Drug Targets.

[14] Tiwari A, Swapna M, Ayesha S, Zehra A, Agawane S, Madhusudana K (2011). Identification of proglycemic and antihyperglycemic activity in antioxidant rich fraction of some common food grains. Int Food Res J.

[15] Wadkar K, Magdum C, Patil S, Naikwade N (2008). Antidiabetic potential and Indian medicinal plants. J Herb Med Tox.

[16] La Regina G, Bai R, Rensen W, Coluccia A, Piscitelli F, Gatti V (2011). Design and synthesis of 2-heterocyclyl-3-arylthio-1h-indoles as potent tubulin polymerization and cell growth inhibitors with improved metabolic stability. J Med Chem.

[17] Jacobo-Herrera NJ, Jacobo-Herrera FE, Zentella-Dehesa A, Andrade-Cetto A, Heinrich M, Pérez-Plasencia C (2016). Medicinal plants used in Mexican traditional medicine for the treatment of colorectal cancer. J Ethnopharmacol.

[18] Michel C, El-sherei M, Islam W, Sleem A, Ahmed S (2013). Bioactivity-guided fractionation of the stem bark extract of *Pterocarpus dalbergioides* Roxb. ex Dc growing in Egypt. Bull Fac Pharm Cairo Univ..

[19] Cheung L, Cheung PC, Ooi VE (2003). Antioxidant activity and total phenolics of edible mushroom extracts. Food Chem.

[20] Chang C-C, Yang M-H, Wen H-M, Chern J-C (2002). Estimation of total flavonoid content in propolis by two complementary colorimetric methods. J Food Drug Anal.

[21] Sun B, Ricardo-da-Silva JM, Spranger I (1998). Critical factors of vanillin assay for catechins and proanthocyanidins. J Agric Food Chem.

[22] Alali FQ, Tawaha K, El-Elimat T, Syouf M, El-Fayad M, Abulaila K (2007). Antioxidant activity and total phenolic content of aqueous and methanolic extracts of Jordanian plants: an ICBG project. Nat Prod Res.

[23] Drent M, Larsson I, William-Olsson T, Quaade F, Czubayko F, Strobel W (1995). Orlistat (Ro 18–0647), a lipase inhibitor, in the treatment of human obesity: a multiple dose study. Int J Obes Relat Metab Disord.

[24] Sudha P, Zinjarde SS, Bhargava SY, Kumar AR (2011). Potent α-amylase inhibitory activity of Indian Ayurvedic medicinal plants. BMC Complement Altern Med.

[25] Ademiluyi AO, Oboh G (2013). Soybean phenolic-rich extracts inhibit key-enzymes linked to type 2 diabetes (α-amylase and α-glucosidase) and hypertension (angiotensin I converting enzyme) in vitro. Exp Toxicol Pathol.

[26] Mosmann T (1983). Rapid colorimetric assay for cellular growth and survival: application to proliferation and cytotoxicity assays. J Immunol Methods.

[27] Afolayan A, Jimoh F, Sofidiya M, Koduru S, Lewu F (2007). Medicinal Potential of the Root of Arctotis arctotoides. Pharm Biol.

[28] Tundis R, Loizzo M, Menichini F (2010). Natural products as α-amylase and α-glucosidase inhibitors and their hypoglycaemic potential in the treatment of diabetes: an update. Mini Rev Med Chem.

[29] Zhang B, Deng Z, Ramdath DD, Tang Y, Chen PX, Liu R (2015). Phenolic profiles of 20 Canadian lentil cultivars and their contribution to antioxidant activity and inhibitory effects on α-glucosidase and pancreatic lipase. Food Chem.

[30] Corina D, Delia M, Ersilia A, Claudia F, Istvan O, Andrea B (2019). Phytochemical characterization and evaluation of the antimicrobial, antiproliferative and pro-apoptotic potential of Ephedra alata Decne. Hydroalcoholic extract against the MCF-7 breast cancer cell line. Molecules.

[31] Šamec D, Maretić M, Lugarić I, Mešić A, Salopek-Sondi B, Duralija B (2016). Assessment of the differences in the physical, chemical and phytochemical properties of four strawberry cultivars using principal component analysis. Food Chem.

